# CIRS-LAS – a novel approach to increase transparency in laboratory animal science for improving animal welfare by reducing laboratory animal distress

**DOI:** 10.3389/fvets.2023.1155249

**Published:** 2023-06-21

**Authors:** Astrid Enkelmann, Sabine J. Bischoff

**Affiliations:** Animal Welfare Office, University Hospital Jena, Friedrich-Schiller University, Jena, Germany

**Keywords:** critical incident reporting system, 3R principle, transparency, laboratory animal science, incidents

## Abstract

The 3Rs principle is highly topical in animal-based research. These include, above all, new scientific methods for conducting experiments without an animal model, by using non-animal models (Replace), reducing the number of laboratory animals (Reduction) or taking measures to keep the stress on the laboratory animal as low as possible (Refinement). Despite numerous modern alternative approaches, the complete replacement of animal experiments is not yet possible.

The exchange in the team about the daily work with laboratory animals, about open questions and problems, contributes to a reflection of one’s own work and to a better understanding of the work of the others. CIRS-LAS (Critical Incident Reporting System in Laboratory Animal Science) represents a reporting system for incidents in laboratory animal science. It is urgently needed because the lack of transparency about incidents leads to the repetition of failed experiments. Negative experiences from animal-based experiments are often not mentioned in publications, and the fear of hostility is still very high. Therefore, a constructive approach to errors is not a matter of course. To overcome this barrier, CIRS-LAS was created as a web-based database. It addresses the areas of reduction and refinement of the 3Rs principle by providing a platform to collect and analyze incidents. CIRS-LAS is open to all individuals working with laboratory animals worldwide and currently exists with 303 registered members, 52 reports, and an average of 71 visitors per month.

The development of CIRS-LAS shows, that an open and constructive error culture is difficult to establish. Nevertheless, the upload of a case report or the search in the database leads to an active reflection of critical occurrences. Thus, it is an important step towards more transparency in laboratory animal science. As expected, the collected events in the database concern different categories and animal species and are primarily reported by persons involved in an experiment. However, reliable conclusions about observed effects require further analysis and continuous collection of case reports. Looking at the development of CIRS-LAS, its high potential is shown in considering the 3Rs principle in daily scientific work.

## 1. Introduction

What are the similarities between animal testing and flight safety, military operations, or production in mechanical engineering? All of these areas benefit from a constructive error culture to optimize processes and prevent the repetition of failed procedures (1). However, an important difference between animal experimentation and these other areas are the possible consequences of failures. In animal experiments, the focus is on the welfare of laboratory animals, which is potentially affected by failures. Despite the development of numerous modern and promising alternative models, animal testing cannot be completely replaced from today’s perspective. In 2021, 2.5 million laboratory animals were used for experimental studies or teaching in Germany (10.6 million in the EU in 2019) (2, 3). Since the EU Directive EU 2010/63 (4) came into force the 3Rs principle (Replace, Reduce, Refine) which had been published long before has been given a legal basis (5).

In Germany as in all other European states the EU Directive EU 2010/63 is applied within the legal regulations for laboratory animal science by the Animal Welfare Act (TierSchG) and the Animal Welfare Ordinance (TierSchVersV) (6, 7). Both legislative texts were extended by the 3Rs principle (3Rs) in 2013. They form the basis of good scientific practice in animal experimentation. According to the 3Rs, all animal experiments should be designed to minimize the number of animals used for experimental purposes (Reduce) and to improve the conditions for the experimental animals (Refine). Whenever possible, alternative approaches must be used if the experimental purpose can also be achieved in this way (Replace). There is a growing awareness not only of working according to the 3Rs principle, but also of the impact of animal welfare on the reproducibility, reliability, and implementation of data obtained from animal experiments (8, 9).

Since 2016 the focus of initiatives and actions was mainly on animal welfare. In the 2020s, the perspective has changed and the wellbeing of people working with laboratory animals is also coming to the fore. The concept of what is known as Culture of Care (CoC) describes good communication as an essential tool to achieve appreciation of laboratory animals and the people who work with them daily (10, 11). Communication in animal experimental research plays an important role at all levels - on the one hand, internally - from the animal caretaker to the management level - and on the other hand, it should be directed externally. External communication primarily involves exchanges with the public about animal experiments and their acceptance. This acceptance is mainly achieved by supplying all the necessary information about animal testing in general (12) and transparency about the exact goal of an animal test (13). Acceptance is further promoted by the support of the European Animal Research Association EARA (14). EARA offers training for researchers toward open communication on animal testing. Institutions from 20 different European countries are member of EARA to work according to the basic principle of transparency in scientific work.

In addition to the information about animal testing available on several Internet platforms mentioned above, scientific institutions around the world are increasingly changing their communication strategy toward greater transparency about specific scientific results obtained in-house with animal testing (15). The internal communication already mentioned above includes both the worldwide exchange with the entire scientific community of people in the field of animal experimentation and the exchange within a scientific institution. The discussion in the team about the daily work with laboratory animals, difficulties and problems, contributes significantly to the reflection of one’s own work and to a better awareness of possible sources of failures (16). However, issues such as the occurrence of an incident or a failure during an experiment, or even the unexpected death of an animal, are often not adequately discussed or even addressed.

For this reason, the desire for a higher level of transparency has come to the fore in recent years. The transparent handling of animal experiments and especially of unexpected incidents is demanded more and more. However, this demand is confronted with mistrust, fear of consequences, and insufficient error awareness in laboratory animal science (LAS). To overcome this mistrust, a confidence base must be established by means of open and constructive communication. At the same time, the advantages of transparency and an open approach to handle errors or incidents must be made clear.

Recognizing errors, discussing the reasons and thinking about possible improvement measures in exchange with others leads to a changed awareness of failures. Mistakes occur, and it is important to accept them and learn from them. In LAS, this means that improvement measures lead to improved animal welfare by avoiding the repetition of unsuccessful experiments. At the same time, dealing openly and constructively with incidents and mistakes is important for building public trust (17). This breaks the cycle of failure and fear of consequences.

Addressing failures constructively goes back to the so-called Swiss cheese model, originally described by the British psychologist James Reason (18). The model represents how latent and active human errors contribute to the breakdown of complex processes and describes the concatenation of error causes. The model compares safety systems with cheese slices placed one behind the other. The holes in the cheese represent the imperfection of safety measures in processes. An unfavorable combination of individual multifactorial defects may cause damage, accidents, or serious consequences. Nowadays, the Swiss cheese model is used worldwide in various disciplines for the analysis of accident causes, in risk analysis, and in risk management. So-called CIRS (critical incident reporting systems) already exist in numerous technical application areas outside medicine, highlighting the importance and suitability of constructive error culture systems (1).

The development towards a better understanding of errors and the rising awareness of the positive impact of revealing and verbalizing errors and pinpointing their causes took place in human medicine as early as the 1980s. This subsequently led to the introduction of error reporting systems in hospitals in 2013 (19). Since the amendment of the German Patient’s Rights Act in 2016 (20), every hospital must implement a CIRS to minimize risks to patient well-being (21, 22).

Based on the experiences of CIRS from human medicine, CIRS was established for LAS in 2015 (23). Several reports on the need for transparent and constructive error management have been published in LAS (24–26). Researchers are increasingly encouraged to mention adverse effects of animal experiments in publications to increase reproducibility (27). However, the use of concrete error management systems is not common in LAS. The few existing systems focus on local error management within a facility, usually as part of a quality management system (24, 28). In these local failure management systems, the focus is primarily on organizational or constructive incidents that are facility-related and confined to one research institution and are therefore very valuable in evaluation. Therefore, the main approach of the reporting system named here, CIRS-LAS, was to create a supra-regional, web-based error management system that is easily accessible for all those involved in animal research. It should not be limited to a single institution, but rather make incidents available to the entire scientific community. The goal of CIRS-LAS was to develop a global approach to network critical incident reports that subsequently could be implemented on an individual research setting.

At,^1^ anyone involved in LAS can enter a critical incident using the case report form without prior registration. Furthermore, the visitors of the website can inform themselves about the project. Research within the case report database is possible after registration at^2^ with name and institution. Registered users can leave comments and suggestions for improvement regarding other reported cases, or read about one’s own registered cases.

The entry of a critical event and thus the provision of information represents a transparent handling of errors and incidents in animal science. Addressing refinement activities through open dialog is important, as it consequently contributes to improved animal welfare. At the same time, it allows for improving the quality of results of scientific studies by minimizing sources of bias such as unexpected events, interference from suffering animals, or other circumstances.

The benefits of CIRS-LAS are sustainable but develop slowly, since it depends heavily on the acceptance of the project within the scientific community. Investing time in this voluntary work for animal welfare draws on the limited time available to scientists and therefore might negatively affect the compliance to report.

CIRS-LAS supports work according to the 3Rs ‘Reduce’ and ‘Refine’ in several ways. It serves as a platform for analyzing the causes of an incident, which is only possible if the critical incident is the subject of discussion and transparent reflection. It also provides the means to share insights with other scientists, and thus can encourage everyone’s willingness to learn from mistakes in animal experiments. Each individual can contribute to reduce the number of laboratory animals by searching the CIRS-LAS database for review reports of similar critical incidents or problems and possible resolution strategies to prevent recurrence. Evaluating critical events in animal experiments, facilitates developing potential solution strategies and refinement methods for one’s own planned experiments.

The goal of CIRS-LAS is to sustainably improve quality and transparency in all daily work with laboratory animals. This daily work includes not only experimental setups, but also animal husbandry and breeding, as well as daily routine of animal handling and teaching of experimental techniques.

## 2. Development of CIRS-LAS

The homepage^3^ was launched in 2015 and contains a database for critical incidents in LAS. A critical incident includes all processes in which an unanticipated event occurs. This event can be the unforeseen death of an animal, but also an unexpected injury or in general, a result that was not expected in this way. Such a critical incident can occur in any field of LAS: in husbandry, breeding or during an animal experiment.

CIRS-LAS started as a project for scientists working with laboratory animals and was soon extended to all people involved in animal experiments, e.g., animal caretakers, technicians or animal welfare officers. The homepage is divided into two parts - an interface visible to all visitors and a user-restricted area visible only to registered users. The open access interface provides the case report form and general information about the project. The user-restricted area allows for case research and includes commenting options. The web-based application and its availability in English, French and German allows access to CIRS-LAS worldwide. Any person without registration can report a critical incident anonymously on the homepage. The case report form requests 4 important contents (see attachment): (1) Assignment of a title and keywords for later search, (2) details of the animal (s) involved, (3) details of the critical incident itself (subject area, background information, description of the critical incident, possible reasons and suggestions for improvement) and (4) details of the reporter (scientist, employee). All entered data will be used for the later statistical analysis.

In the third part of the case report form (details of the critical incidents itself) the incident is categorized. The categories are based on the German legislation on the number of animals used in scientific approaches (29) and, in addition, some categories have been added to cover the whole field of LAS. These include, for example, anesthesia, musculoskeletal system, genetics and breeding, regulatory and non-regulatory purposes, animal husbandry/hygiene/nutrition, or new animal facility construction. Information about the content of the planned experiment is important for understanding the case report and helps to place it in one’s own work. The exact description of the incident shows the deviation from the planned intention. Negative experiences or negative results of an experiment can be specified. The more detailed the background and the incident are described, the easier it is for other registered users to suggest possible improvement measures.

Furthermore, the degree of distress to the animal, if applicable and estimable, is inquired to capture the impact of the critical incident on the animal. The final question on the reported case includes the factors that may have contributed to the incident. Multiple entries are possible, as there are often multiple factors involved (see also Swiss Cheese Model) (18). Here, contributing factors such as organizational problems within the institution itself or equipment and technical failures but also personal factors such as lack of communication, human error, or problems with a special medication as well as factors related to the animal itself can be mentioned.

The complete case report will be checked for anonymity and plausibility by the project administrators before it is published in the user-restricted area of CIRS-LAS. Named keywords are entered into the database (and modified or added if necessary) to facilitate later searches corresponding to an area of interest. To access the restricted database area, registration with a professional e-mail address is required. The CIRS-LAS administrator team manually checks the assignment to the specified institution of the registering persons. The registration will be activated if the institution is conducting animal experiments or if the person can prove a professional interest in LAS. After login, registered users can read their own reports or cases reported by others in the restricted user area of the database. They can also make comments on case reports, which are checked for plausibility and content by the project administrators before being published.

All collected data from registered case reports are statistically evaluated regarding frequency of an animal species, a category, an influencing factor, a reporting group of persons or the influence of an incident on the further course of the experiment or on the severity of an injury. Statistical analyses are performed in Excel due to the number of case reports and the research question.

The desire and willingness for more transparency in dealing with animal experiments and critical incidents has increased greatly in recent years. The increased interest in transparency shows, that there is an urgent need to introduce an incident reporting system. Nevertheless, after the introduction of CIRS-LAS in 2015, the willingness to use the database to report a critical incident was initially low, comparable to the similarly delayed acceptance of CIRS in human medicine.

### 2.1. Increasing acceptance of CIRS-LAS

**Figure 1:**
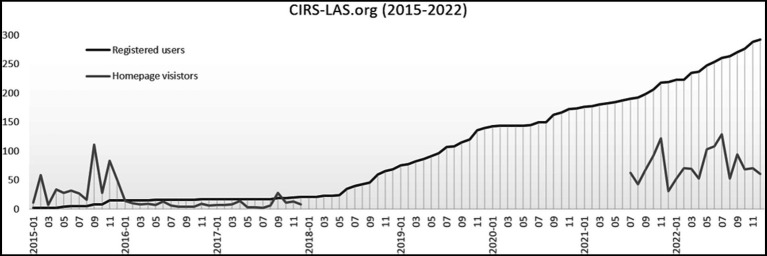
Number of registered users at CIRS-LAS from January 2015 to December 2022; Number of homepage visitors at CIRS-LAS.org from January 2015 to December 2017 and in a tested period of 18 months from July 2021 to December 2022 only includes accesses by unique real users, accesses by bots are not included.

At the beginning of 2015, the trend towards more transparency in LAS was far from being evident and the project was only known in the local area where the project originated. There was still enormous reluctance to use CIRS-LAS as means to handle critical incidents transparently in animal-based research. As a result, the number of people registered increased slowly and required much discussion and persuasion (Figure 1). However, presentations at universities and research institutions, and discussions with people involved in animal research led to an increase in registrations, which are no longer just local, but also national and international. These presentations provided an opportunity to critically discuss CIRS-LAS with researchers, students, technical assistants, and others who work with laboratory animals. Questions about anonymity, benefits, the reporting process, and how to deal with authorities as well as opponents of animal experimentation were answered and discussed. Concerns about anonymity, consequences and even penalties were often dispelled. The intention was to awaken fundamentally their understanding of the need for more transparency in the daily scientific work.

Compared to the beginning, the number of homepage visitors decreased, while more registered users were counted (Figure 1). This may be the result of a more targeted visit to register on see foot note text 3. On the one hand, possible reasons for the initially very hesitant application of CIRS-LAS can be seen in the fact that the trend towards more transparency in animal research developed more strongly only later. On the other hand, the acceptance of CIRS in human medicine developed similarly slowly, since an open-minded error culture had not yet been fully established. Consequently, in the critical discipline of animal-based research, the new approach toward more transparency took even longer.

### 2.2. Analyses of case reports

**Figure 2:**
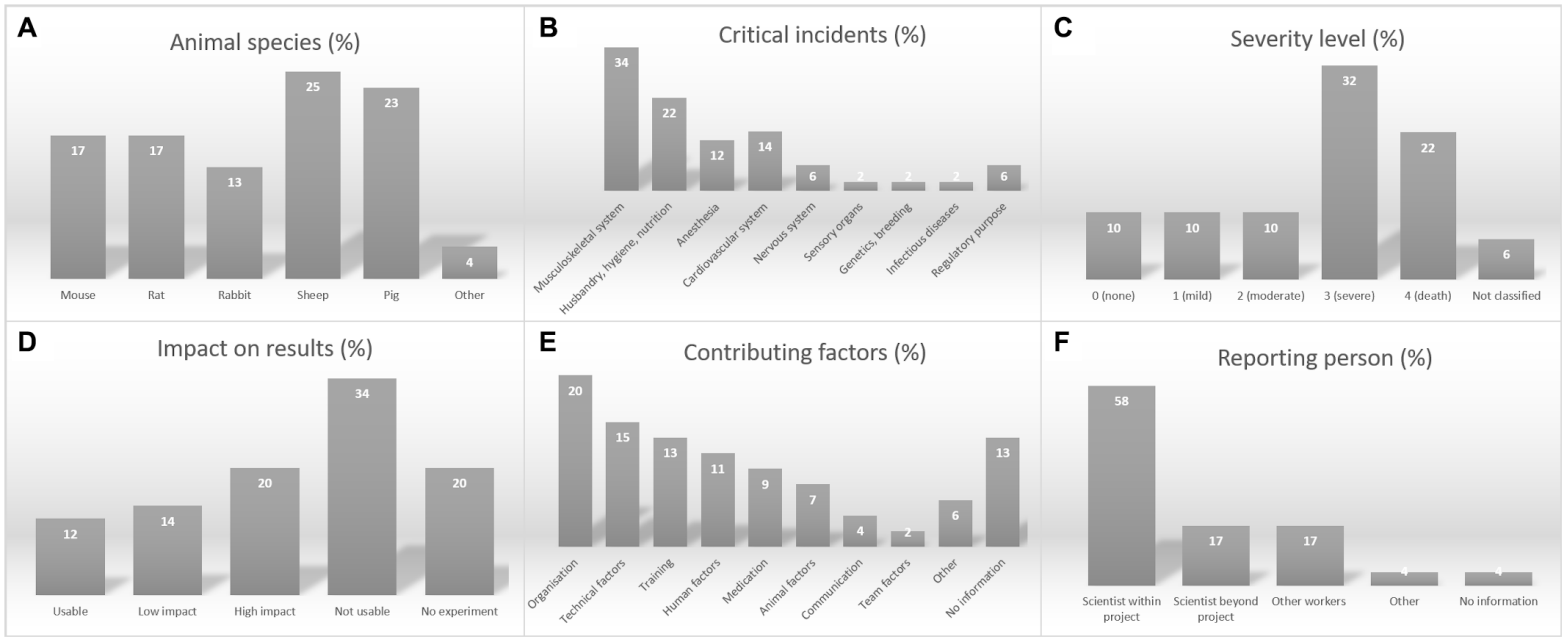
Analysis of the reported incidents in the CIRS-LAS database (*n* = 52); **(A)** Animal species, **(B)** Number of critical incidents in the different disciplines, **(C)** Severity level, **(D)** Impact of the incidents on experimental results, **(E)** Contributing factors, and **(F)** Reporting person; the percentage of cases and the linked information refer to the period of project launch in January 2015 to December 2022.

The number of case reports (*n* = 52) and the fact that each reporting person filled in almost all required fields allowed a statistical validation regarding different aspects of a critical incident report (Figure 2).

Noticeable is the high number of farm animals like sheep and pigs in the reported incidents, followed by rodents (mice, rats) which make up more than 80% of all laboratory animals (Figure 2A). Regarding the incident discipline and the context, most cases were reported in the musculoskeletal field and in laboratory animal husbandry, including the hygiene and nutrition field (Figure 2B). The high number of reported cases concerning farm animals could be based on several possible explanations. First, farm animals are usually not commercially bred homogeneous animals, which is why they are usually not comparable with standardized commercially bred laboratory rodents. Farm animals, usually originated from farm animal environment and thus might be associated with irregular hygienic status. In contrast to laboratory rodents purchased from highly standardized and strictly monitored commercial breeding facilities, their hygienic status is oftentimes not even fundamentally analyzed. Subclinical infections might lead to increased biological variability as well as higher risk of occurring side effects under experimental conditions (30, 31). Secondly, farm animals are used as models for particularly challenging complex models, e.g., in musculoskeletal research to determine critical size effects in bone healing or as models for heart failure (32, 33). Considering these risk factors (farm animal species and difficult research areas) in conjunction suggests a higher risk for incidents here.

The so-called expected severity level (Figure 2C) is determined when applying for an animal experiment permit, i.e., before the experiment begins. The maximum severity level to which an animal is expected to be exposed in the planned experiment is defined in 4 severity levels according to EU Directive 2010/63 (4): low, moderate, severe, non-recovery. The severity of the reports in the CIRS-LAS database was primarily severe, and a high number of critical incidents also resulted in the death of laboratory animals (Figure 2C). The reason for the high number of dead animals and severe courses due to an incident may also be related to the high number of reports with large animals as described in the previous section. In addition to the disciplines of musculoskeletal system and husbandry/hygiene/nutrition already mentioned above in connection with farm animals, anaesthesia and cardiovascular interventions also carry very high risks (Figure 2B). Incidents in these disciplines can more quickly lead to a severe severity level. This is also reflected in the proportion of high impact of an incident on the experiment since most frequently, results could not be used for evaluation or experiments could no longer be used continued (Figure 2D).

It can also be cassumed that a large proportion of incidents are not reported. It is clear however, that reported incidents can have a significant impact on an experiment and, especially when considered on the scope of an entire research facility, can help to avoid a significant number of unsuccessful experiments. Based on the evaluation of CIRS-LAS, it becomes clear that the impact of an efficient error management must not be neglected. Not only the number of laboratory animals that are needed for the repetition of a failed experiment must be taken into consideration, but also the resulting costs and the additional time invested. All these things are precious goods at universities and research institutions.

The reporting persons were also asked for the factors that led to the critical incident. The most frequently stated factor was the organization (Figure 2E) which refers not only to the organization of the experiments, but also to the organizational structure of the facility. This mentioned factor can stand for a lot of different causes and could also be a result of the cumulative effect of several influences, like it is described in the Swiss cheese model (18). Technical factors such as equipment failure or technical malfunctions also led to many reported critical incidents. Several failures occurred in training situations or were caused by human factors such as inattention, fatigue, or lack of motivation, to name a few examples.

The statistical analysis of the reporting persons clearly shows that mostly the scientists involved in a project, and less often external scientists or technical staff, reported the incidents (Figure 2F). Project related scientists are mainly responsible for the wellbeing of laboratory animals used for their experimental purposes. That might be a reason why they reflect and evaluate every critical incident. Their aim is to provide good laboratory science, which is why they often critically question themselves, and their work, and search for transparent published data. The trend towards good laboratory practice has been in place for several years, which has led to increased publication of possibilities for ensuring the reproducibility of experiments.

Regarding other measures to improve the reproducibility of animal experiments, such as the ARRIVE guidelines (34), one study found that in the more than 230 examined publications, none of them fulfilled 100% of the requirements of these guidelines. Five years after the ARRIVE guidelines were published, the quality of the information provided in publications on animal experiments was still not improved across the board (35).

## 3. Discussion

In an era of transparency initiatives and rising interest from the public in animal experimentation and the wise use of public research funds for basic medical research, every institution should think about how it handles incidents and errors in laboratory animal science and should use CIRS-LAS to do so. To successfully improve transparency in animal experiments in all areas, the use of a CIRS must clearly be supported by the management level. Only then, it is possible to give all employees sufficient security and confidence to talk about incidents without fear of sanctions.

The results clearly show that the majority of reported critical incidents occur in the domains of very complex animal models as musculoskeletal experiments as well as in laboratory animal husbandry. The outcome of incidents with respect to severity assessment was mainly named as severe. However, that conclusion can only be regarded as a presumption based on the number of critical incidents referred to the CIRS-LAS database. Compared to evaluation of use of CIRS in human medicine, the majority of reported critical incidents in hospitals occurred in emergency units, intensive care units, and post anaesthesia care units, which suggests that the probability of a critical incident to occur can be associated with high-risk domains in general.

In the case of critical incidents in LAS, the causes can be traced back to human factors and the surrounding organizational structure in the laboratory or institution. A second consideration involves the difficulty of admitting personal failures. Therefore, it might be possible that contributing organizational factors also include personal failure. Reflecting on one’s own work and the mistakes made in the process is still the biggest obstacle, along with the fear of sanctions from colleagues or management. One consequence of this is an unwillingness to enter critical incidents into the CIRS-LAS database. This is also the weakness of CIRS-LAS, which can only be countered by constantly reminding of the goals of an error culture: the improvement of animal welfare and to increase transparency. Both should be in focus of every person who performs animal experiments.

Regarding the question of a local or a global acting reporting system, it should be clear that few incidents are to be expected in a single facility and the exchange about possible reasons and improvement measures is limited. Furthermore, depending on the type of laboratory animals used, not every animal species or category can be mapped. For this reason, CIRS-LAS was designed as a web-based, globally usable system. Only a comprehensive database, representing as far as possible all categories of laboratory animal science, can lead to learning from the mistakes of others. A database with continuously expanding number of cases increases the probability of researching incidents about failed attempts, problems, and possibilities for improvement that affect one’s own field of activity. CIRS-LAS is a platform for anyone who is willing to work transparently and to establish a positive error culture in laboratory animal science. Furthermore, this should provide an opportunity for constructive exchange, because ultimately, animal welfare and the reduction of laboratory animals must come first.

From today’s perspective, it can be concluded that advantages of using CIRS-LAS to improve quality within animal experiments and to reduce numbers of used animals are proved and undeniable. The active implementation of a constructive failure culture is fundamental to scientific progress while maintaining the highest standards of animal welfare. Recognition of the need for transparent communication in the sense of a positive ‘Culture of Care’ will ensure public confidence in laboratory animal science.

## Data availability statement

The raw data supporting the conclusions of this article will be made available by the authors, without undue reservation.

## Author contributions

SB and AE: conceptualization, writing—original draft preparation, review, editing, and validation. SB: methodology, investigation, supervision, project administration, and funding acquisition. AE: formal analysis and visualization. All authors have read and agreed to the published version of the manuscript.

## Funding

This research was funded by the German Federal Ministry of Education and Research (BMBF) under grant numbers 031L130 and 031L230. During the second funding period, there was a change in the funding number due to the change of the funding body: 161L0230. The funders had no role in study design, data collection, and analysis, decision to publish, or preparation of the manuscript.

## Conflict of interest

The authors declare that the research was conducted in the absence of any commercial or financial relationships that could be construed as a potential conflict of interest.

## Publisher’s note

All claims expressed in this article are solely those of the authors and do not necessarily represent those of their affiliated organizations, or those of the publisher, the editors and the reviewers. Any product that may be evaluated in this article, or claim that may be made by its manufacturer, is not guaranteed or endorsed by the publisher.

## Footnotes

^1^ www.cirs-las.org/report_incident

^2^ www.cirs-las.org/register

^3^ www.CIRS-LAS.org
